# Characterizing Tau Neuropathology Across Brain Regions in Patients Carrying PSEN1 Jalisco Mutation

**DOI:** 10.1002/alz.090990

**Published:** 2025-01-03

**Authors:** Bayla M Breningstall, Evan Abdollahi, Anne Hiniker, Debra Hawes, John M Ringman, Michael S Bienkowski

**Affiliations:** ^1^ Laboratory of Neuro Imaging, Keck School of Medicine, University of Southern California, Los Angeles, CA USA; ^2^ Stevens Neuroimaging and Informatics Institute, Keck School of Medicine, University of Southern California, Los Angeles, CA USA; ^3^ USC Center for Integrative Connectomics, Los Angeles, CA USA; ^4^ Department of Pathology, Keck School of Medicine, University of Southern California, Los Angeles, CA USA; ^5^ Alzheimer’s Disease Research Center, Keck School of Medicine, University of Southern California, Los Angeles, CA USA; ^6^ University of Southern California, Los Angeles, CA USA; ^7^ Memory and Aging Center, University of Southern California, Los Angeles, CA USA; ^8^ Alzheimer's Disease Research Center, Keck School of Medicine, University of Southern California, Los Angeles, CA USA; ^9^ Zilkha Neurogenetic Institute, Keck School of Medicine, University of Southern California, Los Angeles, CA USA

## Abstract

**Background:**

Mutations in the presenilin 1 (PSEN1) gene cause early onset autosomal dominant Alzheimer’s Disease (AD), and the Jalisco mutation (A431E) is a subset found in people of Mexican descent (Yescas P, 2006). The Jalisco A431E mutation has been shown to produce distinct AD neuropathology such as cotton‐wool amyloid plaques as well as motor dysfunction like spastic paraparesis (Orozco‐Barajas, 2022). High levels of tau neuropathology have been observed with other PSEN1 mutations, but tau neuropathology in Jalisco patients has not been examined. Therefore, we sought to quantify the distribution of tau across a variety of brain regions in patients with A431E mutation and compare to patients with other PSEN1 mutations (A260V and T245P) or high stage sporadic AD.

**Method:**

Ten coronal brain sections from 9 PSEN1 (n = 5 A431E; n = 2 A260V, n = 2 T245P) and 9 high stage AD subjects immunohistochemically labelled with AT8 were obtained from the USC Alzheimer’s Disease Research Center (ADRC). Regions analyzed included cerebral cortex (prefrontal, parietal, temporal, and occipital areas), hippocampus, striatum, midbrain, cerebellum, pons, and medulla. Ilastik, a machine‐learning based software, was trained on positive AT8 staining versus background pixels and applied this training to the whole slide image. This binary output was then overlaid with a manually drawn ROI atlas in the Quantitative Imaging Toolkit (QIT), and positive pixels per pixels of the region was used to calculate density of AT8 pathology.

**Result:**

AT8 density was significantly higher across the brain in patients with PSEN1 mutations compared to sporadic AD. Although small sample size is limited for these patient groups, we found that A431E patients had lower AT8 density in the hippocampus compared to other PSEN1 patients, most notably in dentate gyrus, as well as Brodmann Areas 35 and 36.

**Conclusion:**

These data show a high burden of tau fibrils as measured by AT8 across brain regions in people with PSEN1 mutations as compared to other high AD patients. Notably, A431E patients had different distributions than other PSEN1 patients. These findings help further characterize early onset autosomal dominant AD within patients of Mexican heritage and how tau neuropathology might contribute to its unique symptoms.

